# Post-translational modifications of vimentin reflect different pathological processes associated with non-small cell lung cancer and chronic obstructive pulmonary disease

**DOI:** 10.18632/oncotarget.27332

**Published:** 2019-11-26

**Authors:** Neel Ingemann Nissen, Morten Karsdal, Nicholas Willumsen

**Affiliations:** ^1^Biotech Research & Innovation Centre (BRIC), University of Copenhagen, DK-2200 Copenhagen, Denmark; ^2^Nordic Bioscience, Biomarkers and Research, DK-2730 Herlev, Denmark

**Keywords:** vimentin, NSCLC, COPD, liquid biopsies, biomarkers

## Abstract

Introduction: Vimentin has shown to be highly implicated in cancer initiation and progression. Vimentin is often a target of post-translational modifications (PTMs) which can be disease specific, thus targeting these specific modifications can be of high biomarker potential. In this study we set out to evaluate the biological relevance and serum biomarker potential of citrullinated vimentin (VICM) and non-citrullinated vimentin (VIM) in non-small cell lung cancer (NSCLC) and chronic obstructive pulmonary disease (COPD).

Methods: A competitive ELISA targeting VIM was developed and quantified in serum from patients with NSCLC and COPD. VIM was compared with levels of VICM in the same indications.

Results: VIM was significantly increased in NSCLC (*n =* 100) compared to healthy controls (*n =* 67) in two independent cohorts (*p* = 0.0003 and *p* < 0.0001). Furthermore, VIM was highly increased in late stages of NSCLC (*p* = 0.001), however VIM was not increased in COPD patients (*n =* 10). Contrarily, serum levels of VICM was not increased in late stages of NSCLC, but highly elevated in patients with COPD (*p* < 0.0001).

Conclusions: These findings suggest a biomarker potential of VIM in NSCLC. Our findings also indicate that PTMs of vimentin are highly relevant and that targeting these modifications can have differential biomarker potential.

## INTRODUCTION

Precision medicine has rapidly moved to the forefront of cancer research by the application and evaluation of a broad range of molecular biomarkers. Most biomarker research in cancer focus on quantifying genetic alterations within the tumor and tumor cells. Liquid biopsies are increasingly tested as novel cancer biomarkers by evaluating the so-called ‘tumor circulome’ consisting of circulating tumor nucleic acids (ctDNA and ctRNA), circulating tumor cells (CTCs), and tumor extracellular vesicles [1, 2]. However, the potential of evaluating circulating proteins derived from the tumor microenvironment as novel liquid biopsies is undervalued.

Post-translational modifications (PTMs) of proteins can be a result of specific physiological and pathophysiological processes and can therefore, when identified, be used as disease specific markers. PTMs have been shown to reveal so called neoepitopes which, when targeted, have shown high potential as diagnostic and prognostic biomarkers in various cancer diseases [3–9]. Examples of PTMs are cross-linking of protein chains, hydroxylation of prolines, protease-generated fragments creating free ends, glycosylation, sumoylation, O’glcNAc and citrullination [10–15].

Vimentin, a protein which is a target of many PTMs including sumoylation, O’glcNAc and citrullination has recently gained attention due to its involvement in cancer [16–19]. Vimentin is an intermediate filament and its main role is to support cellular architecture and integrity. Besides being a structural protein, vimentin has many roles within cell differentiation and division, cell motility, migration, adhesion and cell death [16, 20–28]. Vimentin is upregulated in various cancer diseases including malignant melanoma, prostate, breast, gastro-intestinal and lung cancer [29–35]. In addition, vimentin is used as a canonical marker of epithelial-to-mesenchymal transition (EMT) and cancer-associated fibroblasts (CAFs) [36–39]. Originally it was thought that vimentin only had intracellular roles, however it is now appreciated that vimentin is also found extracellularly. The data concerning the function of extracellular vimentin are very limited. In the inflammatory setting, extracellular vimentin has been associated with various inflammatory states in different organs such as lupus erythematosus, pulmonary sarcoidosis, idiopathic pulmonary fibrosis and atherosclerosis [40–43]. In the vascular setting, extracellular vimentin has shown to be important in blood clotting via its contribution to Von Willebrand’s factor string formation [44]. Furthermore, in wound healing, extracellular vimentin plays a role in the transition of mesenchymal cells to myofibroblasts, and in mice with spinal cord injury, vimentin induces axonal growth and increases motor function [45–47]. Finally, in the oncology setting, extracellular vimentin has been evaluated in patients with Sezary syndrome, malignant melanoma and lung cancer. Interestingly, in patients with malignant pleural mesothelioma vimentin correlates with disease state [8, 30, 48, 49]. Thus, the relevance of extracellular vimentin is rather unclear and must be further investigated.

During tumor development and progression extracellular vimentin may be a target of PTMs such as proteolytic cleavage by matrix metalloproteases (MMPs) overexpressed in the tumor microenvironment as well as citrullinations [16, 20, 50, 51]. Citrullination is the process in which arginine residues are modified into citrulline in a Ca^2+^ dependent conversion mediated by peptidyl arginine deiminase enzymes, which are often present during inflammatory states [52–55]. Interestingly, citrullinated vimentin has been found elevated in serum from lung cancer patients supporting a rationale for targeting vimentin as a liquid biopsy biomarker in this disease [30]. However, citrullinated vimentin is also highly elevated in patients with various inflammatory diseases [56–60]. To further evaluate the association between vimentin processing/PTMs and lung pathology we measured MMP-degraded citrullinated vimentin (VICM) and the non-citrullinated counterpart (VIM) in serum from two independent cohorts of patients with non-small cell lung cancer (NSCLC), a cohort of patients with chronic obstructive pulmonary disease (COPD) and healthy controls.

## RESULTS

### Technical evaluation of the VIM assay

**Figure 1:**
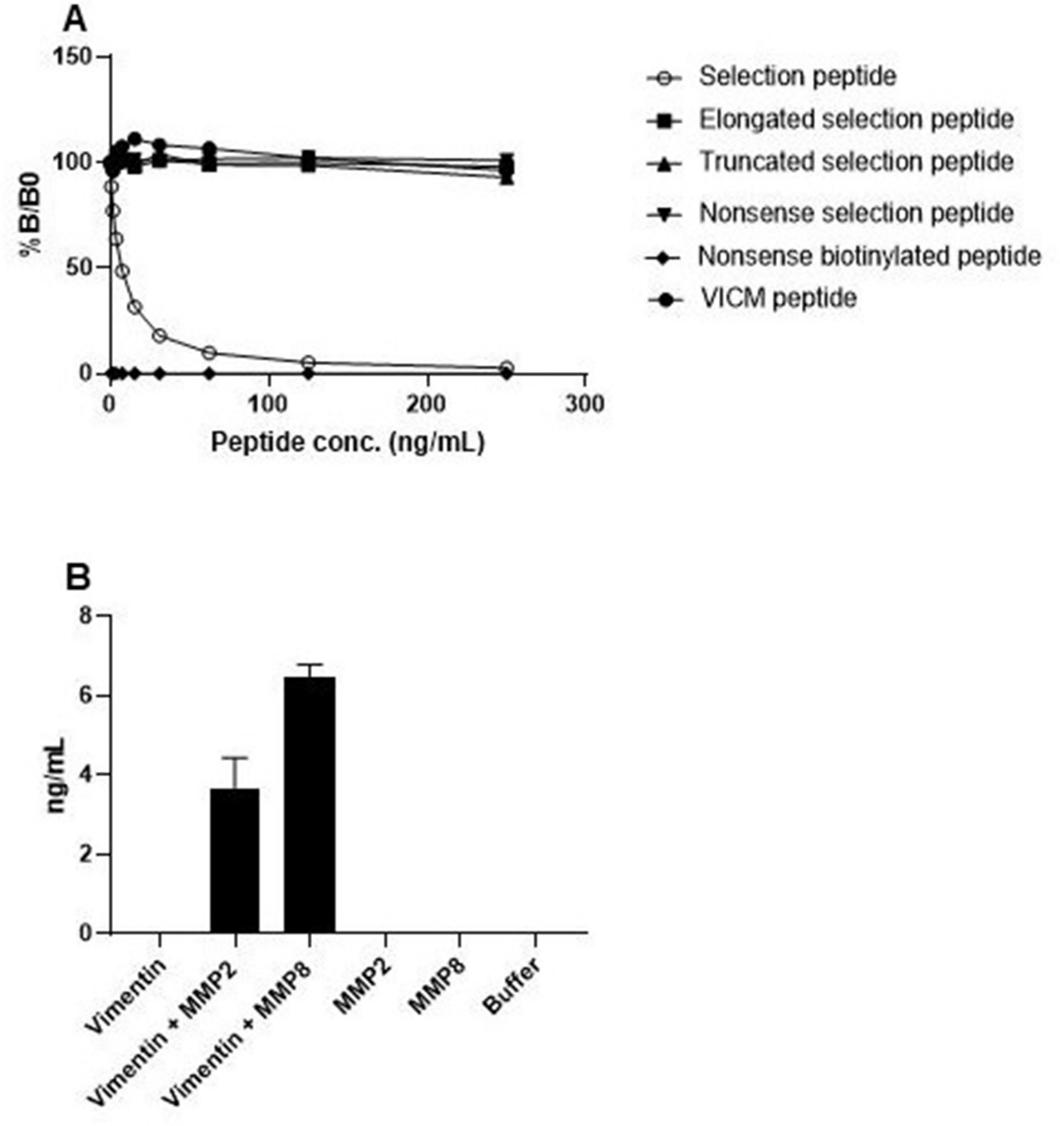
Specificity test of the MMP-degraded non-citrullinated vimentin (VIM) assay. (**A**) The reactivity of the VIM antibody was tested against the selection peptide (RLRSSVPGVR), elongated peptide (RLRSSVPGVRL), truncated peptide (RLRSSVPGV), nonsense peptide (LLARDFEKNY), nonsense biotinylated peptide (LLARDFEKNY-biotin) and the VICM peptide (RLRSSVPGV-Citrulline). %B/B0: B equals the OD at x ng/ml peptide and B0 equals the OD at 0 ng/ml peptide. (**B**) The reactivity of the VIM antibody was tested against recombinant full length vimentin, recombinant full length vimentin cleaved with MMP2 and recombinant full length vimentin cleaved with MMP8.

The specificity of the VIM assay was evaluated by analyzing the reactivity towards the selection peptide (RLRSSVPGVR), an elongated version of the selection peptide (RLRSSVPGVRL), a truncated version of the selection peptide (RLRSSVPGV), a nonsense selection peptide (LLARDFEKNY), a nonsense biotinylated coating peptide (LLARDFEKNY-biotin) and a citrullinated version of vimentin in which arginine is replaced with citrulline (RLRSSVPGV-Citrulline). The antibody only showed reactivity towards the selection peptide with a dose-dependent signal inhibition, suggesting specificity of the antibody towards the selection peptide (Figure 1A).

To further investigate the specificity of the VIM assay, and to ensure that the VIM antibody only detects vimentin fragments generated by MMP2 and MMP8, recombinant full-length vimentin was incubated with the two MMPs. The antibody only showed reactivity towards vimentin fragments cleaved by MMP2 and MMP8, and not the full-length protein, suggesting that the VIM antibody only recognizes vimentin fragments cleaved by the two proteases (Figure 1B).

The overall technical performance of the VIM assay was investigated using the following tests; intra-inter assay variation, dilution recovery, freeze-thaw recovery, analyte stability and recovery in biotin, lipid and hemoglobin. The results are summarized in Table 1. The detection range was determined to 1–132 g/mL (corrected for pre-dilution). The intra-assay variation was 5% and inter-assay variation 11%, and hereby acceptable as the acceptance criterion is below 10 and 15% for the two, respectively. Mean dilution recovery for 1:2 prediluted serum samples was 106%. The serum analyte stability was evaluated by observing analyte recovery after 4 freeze-thaw cycles and analyte storage at 4–20°C for 2–48 hours, with an acceptance criterion of the recovery within 100% ±20%. Spiking of selection peptide in human serum resulted in 100% mean recovery, indicating that the assay response is accurate and not affected by sample matrix. Analyte recovery after freeze-thaw cycles was 94%. Recovery of analyte stored at 4°C for 2–48 hours was 103–116%. Recovery of analyte stored at 20°C for 2–24 hours was 107–80%, thus recovery for analyte stored at 20°C for more than 24 hours could not be reached. This indicates that the analyte is stable up to 48 hours when stored at 4°C, however upon analysis the analyte should not be stored at 20°C for more than 24 hours. With the acceptance criterion at 100% ±20%, there was no detectable interference from biotin, lipid and hemoglobin with recoveries ranging from 87–114%.

**Table 1:** Technical validation of the MMP-degraded non-citrullinated vimentin (VIM) assay

Technical validation step	Results
Detection Range	1–132 ng/mL
Intra-assay variation	5%
Inter-assay variation	11%
Dilution recovery in serum	106%
Freeze-thaw recovery in serum	94%
Spiking Recovery	100%
Analyte stability range in serum 4°C, 2 h–48 h	103–116%
Analyte stability range in serum 20°C, 2 h–24 h	107–80%
Interference:	
Recovery in Biotin low/high	108%/114%
Recovery in Lipid low/high	87%/92%
Recovery in Haemoglobin low/high	96%/99%

Percentages are reported as means.

### Evaluation of VIM in serum from patients with solid tumors

To validate clinical relevance of the VIM assay, it was measured in serum from patients with different solid tumors (colon, lung, melanoma, ovary, prostate, stomach, breast and pancreas) and healthy controls. VIM was highly elevated in all indications suggesting that VIM is relevant in solid tumors (Supplementary Figure 1).

### Serum VIM and VICM in NSCLC compared to healthy controls and patients with COPD

**Figure 2:**
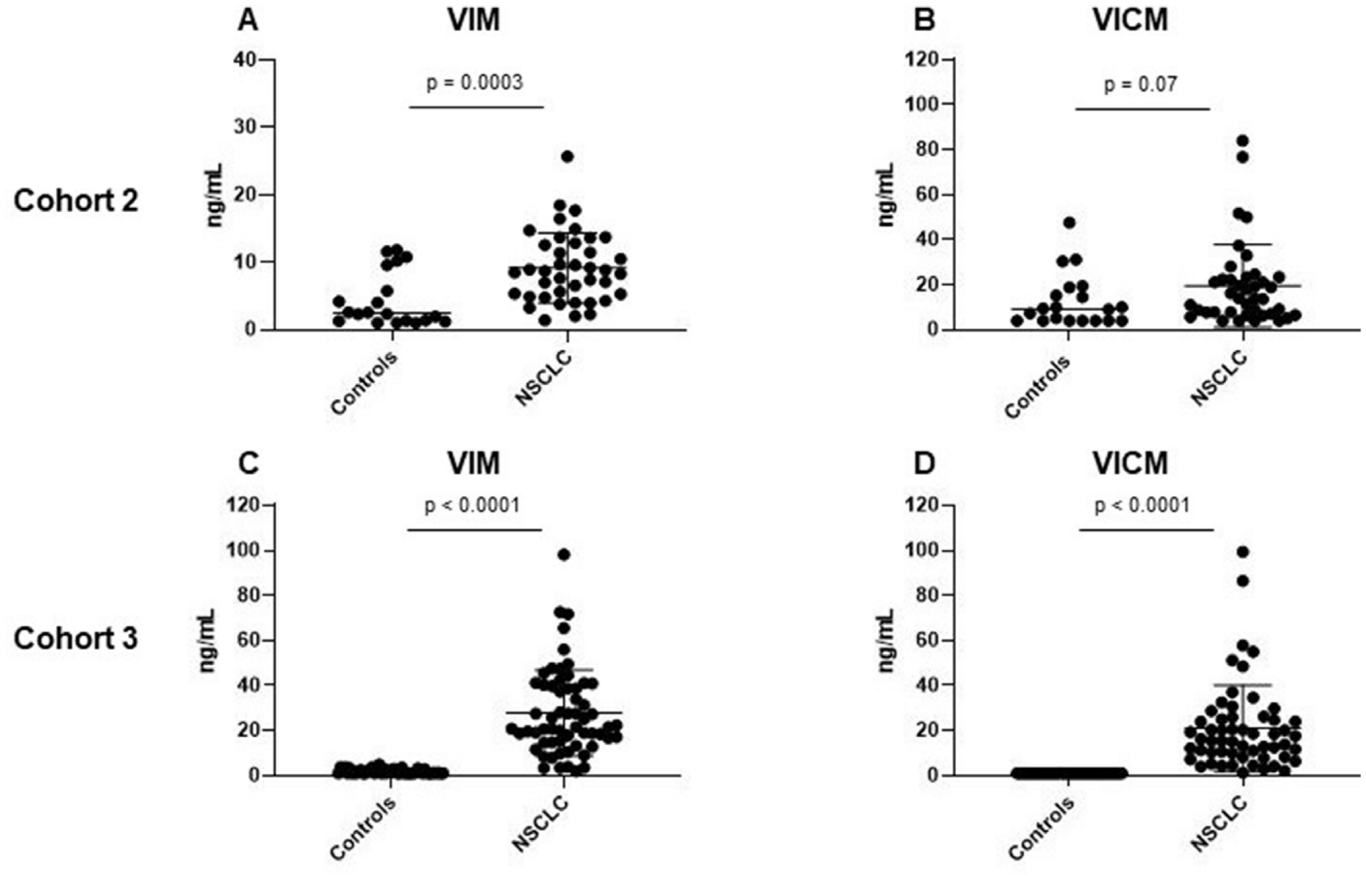
Clinical evaluation of MMP-degraded vimentin (VIM) and MMP-degraded and citrullinated vimentin (VICM) in patients with non-small cell lung cancer (NSCLC). Levels of VIM and VICM in serum were evaluated in two independent cohorts of patients with NSCLC and healthy controls. VIM and VICM levels were compared to each other using the nonparametric Mann-Whitney test. Significance level: *p* < 0.05. (**A**) Evaluation of VIM in cohort 2. (**B**) Evaluation of VICM in cohort 2. (**C**) Evaluation of VIM in cohort 3. (**D**) Evaluation of VICM in cohort 3.

VIM and VICM were measured in serum samples from patients with NSCLC (stage I–IV) and healthy controls. The assays were measured in serum from two independent groups of patients with stage I–IV NSCLC (cohort 2 and 3). The two cohorts were measured at two different time points to validate biomarker levels in two independent cohorts. In cohort 2, consisting of 40 NSCLC stage I–IV patients and 20 controls VIM was significantly increased in NSCLC compared to controls (*p* = 0.003) (Figure 2A). Interestingly, VICM was only slightly elevated with no statistical significance (*p* = 0.07) (Figure 2B). In cohort 3, consisting of 60 NSCLC stage III-IV patients and 47 controls, both VIM and VICM were significantly elevated in NSCLC compared to controls (VIM: *p* < 0.0001, VICM: *p* < 0.0001) (Figure 2C–2D).

**Figure 3:**
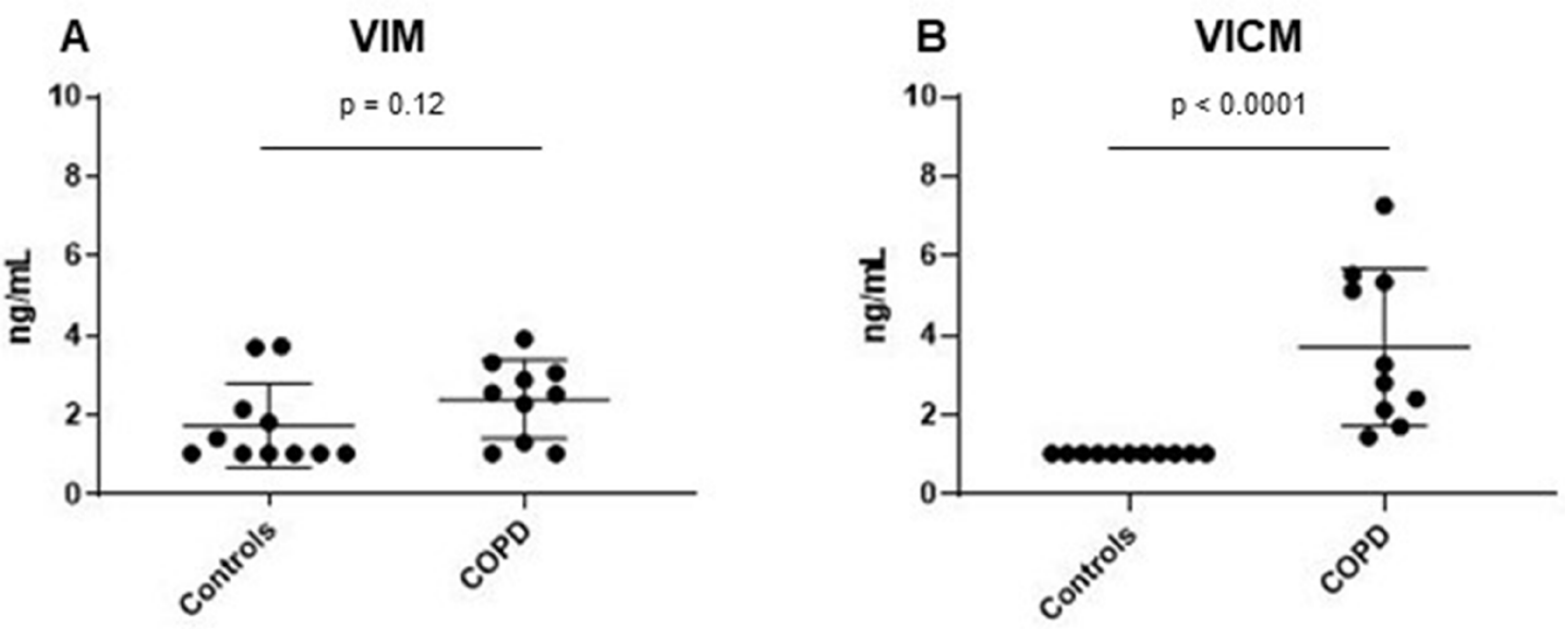
Clinical evaluation of MMP-degraded vimentin (VIM) and MMP-degraded and citrullinated vimentin (VICM) in chronic obstructive pulmonary disease (COPD). Levels of VIM and VICM in serum were evaluated in patients with COPD and healthy controls. VIM and VICM levels were compared to each other using the nonparametric Mann-Whitney test. Significance level: *p* < 0.05. (**A**) Evaluation of VIM in COPD. (**B**) Evaluation of VICM in COPD.

Next, we wanted to investigate VIM and VICM serum levels in a small cohort of COPD patients (*n* = 10), as VICM has shown to be of high relevance in inflammatory diseases [57, 58]. COPD is a chronic inflammatory lung disease which causes obstruction of the airways. Interestingly, there was no difference in serum VIM levels between COPD patients and controls (*p* = 0.12) (Figure 3A). However, VICM was highly elevated in this cohort (*p* < 0.0001) (Figure 3B), suggesting that the two biomarkers are a measure of different pathophysiological phenomena.

### VIM and VICM vs. NSCLC stage

**Figure 4:**
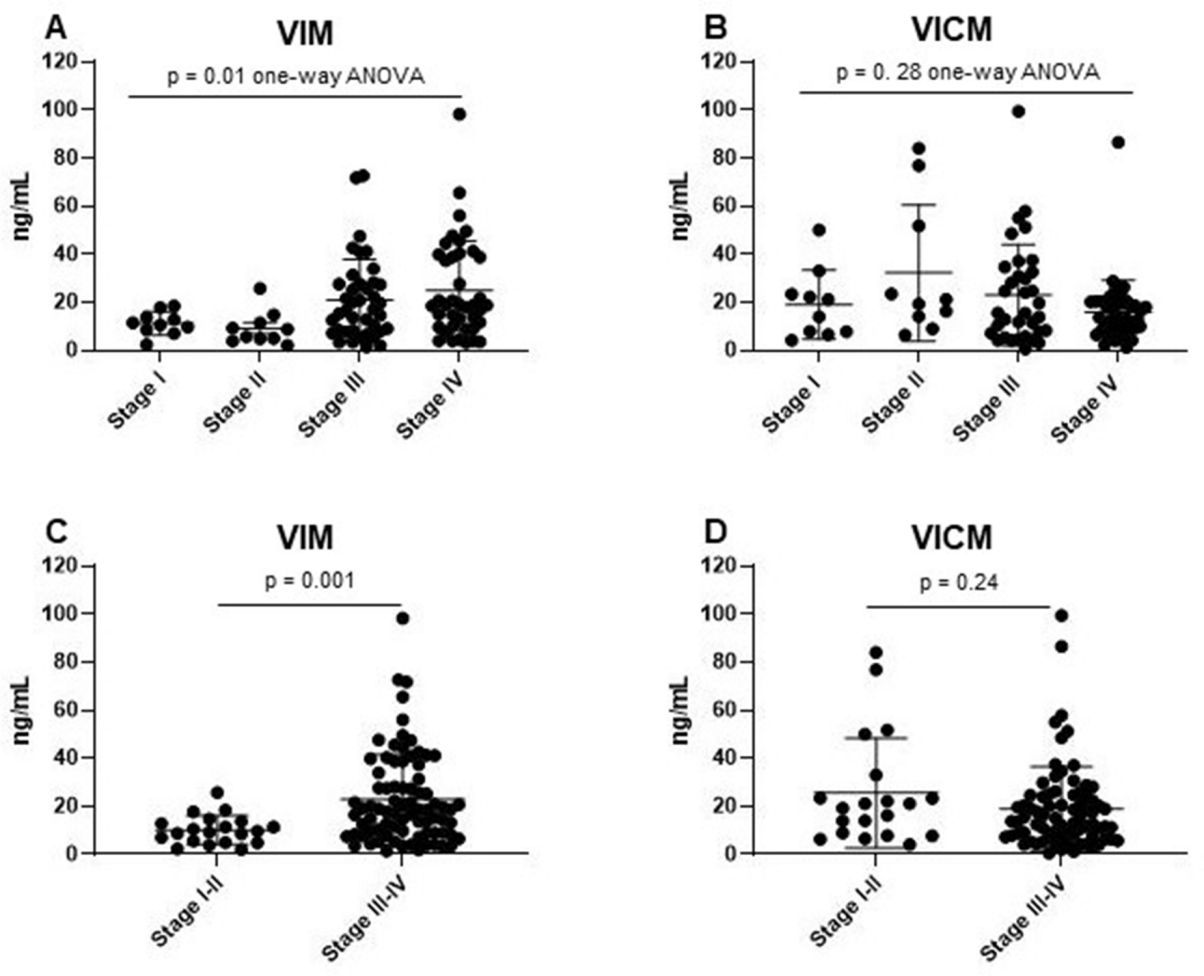
Clinical evaluation of MMP-degraded vimentin (VIM) and MMP-degraded and citrullinated vimentin (VICM) in patients with non-small cell lung cancer (NSCLC) according to TNM disease stage. Levels of VIM and VICM in serum were evaluated in the two groups of patients with NSCLC stratified according to TNM disease stage I, II, III, IV (**A** and **B**) and I–II vs. III–IV (**C** and **D**). VIM and VICM levels were compared to each other using a one-way ANOVA with Kruskal-Wallis test (A and B) and the nonparametric Mann-Whitney test (C and D). Significance level: *p <* 0.05.

The phenotypic distinctions observed between the two biomarkers led us to assess VIM and VICM in different stages of NSCLC. All NSCLC patients, including cohort 2 and cohort 3, were combined and stratified into their respective stages. There was a significant difference of VIM levels between stages of NSCLC (one-way ANOVA *p* = 0.01) (Figure 4A). When combining stage I–II and stage III–IV, VIM was significantly elevated in late stages of NSCLC compared to early stages (*p* = 0.001) (Figure 4C). In contrast, there was no difference in VICM levels between stages of NSCLC (stage I–IV: one-way ANOVA *p* = 0.28, stage I-II vs. stage III–IV: *p* = 0.24) (Figure 4B and 4D).

### Correlation between VIM and VICM

**Figure 5:**
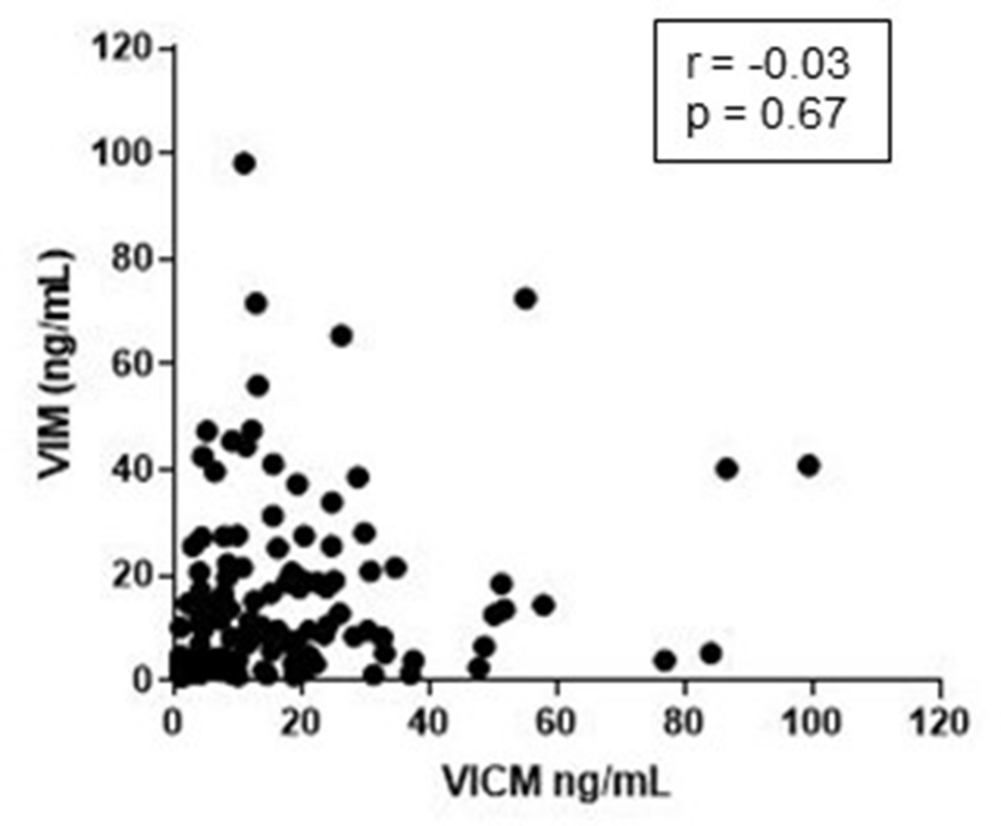
Correlation between MMP-degraded vimentin (VIM) and MMP-degraded and citrullinated vimentin (VICM). Levels of VIM and VICM in serum from patients with non-small cell lung cancer, chronic obstructive pulmonary disease and healthy controls where compared pairwise and evaluated by the Pearson correlation coefficient (r).

To evaluate the extent to which the two variables were linearly related, VIM and VICM were compared pairwise and evaluated with Pearson’s correlation coefficient. The analysis included both the healthy controls, COPD and cancer patients. No correlation between VIM and VICM serum levels was seen (r = –0.03 *p* = 0.67) (Figure 5), emphasizing that the two biomarkers might be a measure of two different pathophysiological events.

### Ability of VIM and VICM to discriminate between NSCLC, COPD and controls (diagnostic power)

**Figure 6:**
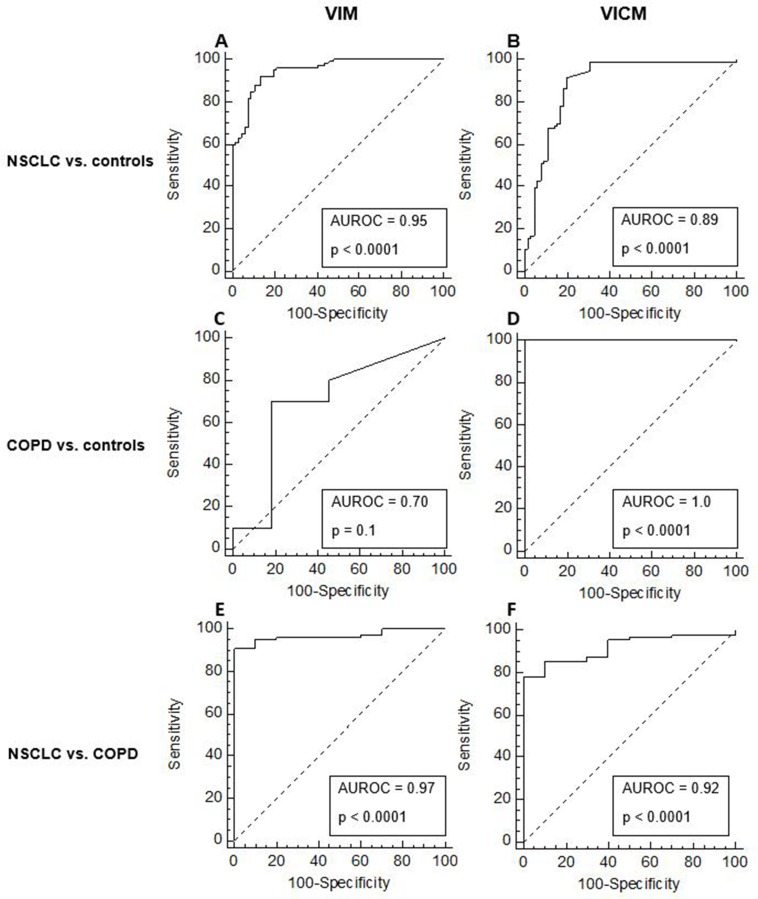
Diagnostic power of MMP-degraded vimentin (VIM) and MMP-degraded and citrullinated vimentin (VICM) for separating non-small cell lung cancer (NSCLC) and chronic obstructive pulmonary disease (COPD) from healthy controls. The diagnostic power of VIM and VICM to discriminate between NSCLC vs. healthy controls (**A–B**), COPD vs. controls (**C–D**) and NSCLC vs. COPD (**E–F**) were calculated using the area under the receiver operating characteristics curve (AUROC).

To investigate the ability of VIM and VICM to discriminate between NSCLC, COPD and controls, the diagnostic power of VIM and VICM was calculated. The diagnostic power was based on the area under the receiver operating characteristics (AUROC) and by comparing NSCLC vs. controls, COPD vs. controls and NSCLCL vs. COPD. When compared head to head, VIM was superior to VICM in terms of separating NSLCL from controls (VIM: AUROC 95%, *p* < 0.0001, VICM: AUROC 89%, *p* < 0.0001) (Figure 6A–6B) and in separating NSCLC from patients with COPD (VIM: AUROC 97%, *p* < 0.0001, VICM: AUROC 92%, *p* < 0.0001) albeit the low n (Figure 6E–6F). However, VICM was superior to VIM in separating patients with COPD from controls (VIM: AUROC 70%, *p* = 0.1, VICM: AUROC 100%, *p* < 0.0001) (Figure 6C–6D).

## DISCUSSION AND CONCLUSIONS

In this study, we evaluated the association of two different PTMs of vimentin, VICM and VIM, in patients with NSCLC, COPD and controls. To our knowledge, this is the first study to suggest that the involvement of vimentin in two distinct pathologies, even within the same organ, might be reflected by specific PTMs. We observed increased serum levels of VIM measured in two independent groups of patients with NSCLC. In addition, the increased levels were even more pronounced in late stages of NSCLC. In contrast, VIM was not increased in serum from COPD patients. When measuring VICM, we observed elevated levels in NSCLC patients but no association with stage of disease. Moreover, VICM was elevated in patients with COPD. Hence, these findings support previously published data which show that vimentin is elevated in COPD and NSCLC [30, 61].

Our findings indicate that two MMP degraded vimentin fragments can measure two distinct pathophysiological events. And as supported by previous research, we observed that VIM is potentially more associated with tumorigenesis and VICM is potentially more associated with general chronic inflammation [30, 56–59, 62].

Elevated levels of vimentin are reported for numerous solid tumor types suggesting a role for vimentin in cancer pathologies [29–35]. These data support our findings which show that VIM is increased in serum from patients with breast, colon, lung, melanoma, ovary, pancreas and stomach cancer. In addition, accumulating evidence shows that vimentin plays a role especially in the progression of NSCLC, which is also in line with our results [16, 20, 63] showing that VIM and VICM are increased in patients with NSCLC compared to healthy controls.

Several studies have shown a contributing role of vimentin in tumor progression and late stages of NSCLC which are characterized by invasion of lymph nodes, chest wall and metastasis to other parts of the body [64]. Vimentin predicts metastases and poor overall survival in NSCLC and the expression of vimentin is associated with tumor cell invasion [65–67]. Vimentin is a regulator of EMT which results in tumor cells being able to invade and metastasize neighboring tissue [34, 35, 38, 39, 63]. Furthermore, the expression of vimentin in CAFs is a requirement in the progression of early stage lung adenocarcinoma [35]. Thus, it can be speculated if these events can be explanations for the increased levels of VIM in late stages of NSCLC. Interestingly, VICM was not elevated in late stages of NSCLC further supporting that VIM is associated with tumor biology and potentially reflects tumor burden.

It has been shown that citrullinated proteins are commonly present in inflamed tissue suggesting that this process is inflammation-dependent [53]. PTMs can regulate the function of vimentin and it can be speculated if they can be a function of a specific pathology. Our results showed that VIM and VICM did not correlate, again suggesting that the two biomarkers are a measure of distinct pathophysiological events. We have previously shown that VICM is highly elevated in inflammatory diseases such as rheumatoid arthritis, ankylosing spondylitis and Crohn’s disease [56–59]. In addition, citrullinated vimentin has been shown to be present in lung tissue from patients with COPD [61]. These findings are in line with our results showing that serum VICM is elevated in patients with COPD. These results further support our hypothesis that VIM is associated with tumor progression, while VICM may be more reflective of common states of inflammation independent of tumor stage. However, it remains to be established whether the differences seen between groups are biologically relevant in terms of a significant pathological event.

The present study is limited by several factors. Firstly, albeit findings are validated in independent cohorts the sample size of patients is relatively small. Secondly, controls and patients in cohort 1, 3 and 4 are not obtained from the same vendor. Furthermore, patients and controls in these three cohorts are not gender, age and ethnicity matched which can have confounding effects. However, results from cohort 2, containing patients matched on gender, age and ethnicity, are similar to the results obtained in cohort 3. Thirdly, the study is limited by the cross-sectional study with limited availability of clinical and pathological characteristics. Hence, it would be desirable to study VIM and VICM in a larger longitudinal study with well described clinical and demographic characteristics.

In summary, in this study we measured VIM and VICM and found different expression patterns in serum from patients with NSCLC, COPD and healthy controls. Overall, VIM was superior to VICM in discriminating NSCLC patients from patients with COPD and healthy controls. In contrast, VICM was more associated with COPD patients than VIM. The findings demonstrate that specific PTMs of vimentin are highly relevant in different pathological settings and that targeting these modifications can have differential biomarker potential reflecting diverse disease states. Future investigations are warranted to fully understand the biomarker potential and underlying mechanisms of vimentin modifications.

## MATERIALS AND METHODS

### Patients

Four patient cohorts were validated in this study. Cohort 1 was obtained from the commercial vendor Asterand Bioscience (Detroit, MI, USA) and included patients with different cancer indications; breast (*n* = 2), colon (*n* = 3), lung (*n* = 12), malignant melanoma (*n* = 6), ovarian (*n* = 8), pancreas (*n* = 4), prostate (*n* = 6) and stomach cancer (*n* = 7) and 19 controls (Valley BioMedical, VA, USA) and. Patients in cohort 2, 3 and 4 were obtained from the commercial vendor ProteoGenex (CA, USA) and controls from Valley BioMedical (VA, USA). Cohort 2 included 40 NSCLC patients (stage I–IV) and 20 controls. Cohort 3 included 60 NSCLC patients (stage III–IV) and 47 controls. Cohort 4 included 10 patients with COPD and 10 controls. Appropriate Institutional Review Board/Independent Ethical Committee approved sample collection and all subjects filed informed consent. Demographics and clinical profiles are shown in Table 2.

**Table 2:** Patient demographics and clinical profiles

Group		No. of patients	Stage				Gender, (% females)	Age, median (range)	Race
			***I***	***II1***	***III***	***IV***			
**Cohort 1**									
	*Cancer*	48	-	-	-	-	56%	61 (30–82)	100% C
	*Controls*	19	-	-	-	-	16%	37 (22–54)	16% C 84% B
**Cohort 2**									
	*NSCLC*	40	10	10	10	10	50%	62 (60–65)	100% C
	*Controls*	20	-	-	-	-	50%	61 (60–65)	100% C
**Cohort 3**									
	*NSCLC*	60	-	-	30	30	43%	61 (58–78)	100% C
	*Controls*	47	-	-	-	-	19%	33 (21–59)	21% C 79 % B
**Cohort 4**									
	*COPD*	10	-	-	-	-	50%	54 (50–60)	100% C
	*Controls*	10	-	-	-	-	20%	28 (22–46)	10% C 91% B

NSCLC, non-small cell lung cancer; COPD, chronic obstructive pulmonary disease; C, Caucasian; B, black.

### Biomarker measurements

Levels of VICM in serum were measured according to manufactures instruction’s (Nordic Bioscience A/S, Denmark). For technical evaluation of the VICM assay see [60]. VIM levels in serum were measured according to the procedures described below.

### VIM assay development

#### Antibody development

The identification of the VIM fragment, antibody production and immunization have been described in detail by our group [60]. Briefly, human cartilage was digested with a range of proteases (MMP2, 3, 8, 9, 12 and 13) and the proteolyzed peptide products were identified by mass spectrometry. For antibody development, ten different vimentin fragments were selected for immunization. All relevant sequences were analyzed for homology and blasted for homology using the NPS@: network protein sequence analysis [68]. Monoclonal antibodies were raised in 6-week-old BALB/c mice [60].

The vimentin sequence RLRSSVPGVR immunization generated the best antibody in terms of native reactivity and stability and was chosen for assay development. The sequence located at amino acid position 69 and 78 was identified as being generated by MMP2 and MMP8. The antibody was tested against reactivity towards the selection peptide (RLRSSVPGVR), an elongated version of the selection peptide (RLRSSVPGVRL), a truncated version of the selection peptide (RLRSSVPGV), a nonsense peptide (LLARDFEKNY) and a citrullinated version of vimentin in which arginine is replaced with citrulline (RLRSSVPGV-Citrulline).

#### VIM ELISA assay

Optimal incubation timer, buffer, temperature and optimal ratio between the biotinylated peptide and antibody were determined. The competitive ELISA procedure was as follows: 96-well streptavidin-coated microtiter plates were coated with 100 ul of 1.5 ng/mL biotinylated peptide (Biotin-K-RLRSSVPGVR) dissolved in assay buffer (50 mM PBS-BTB, 8 g/L NaCl, pH 7.4) and incubated in darkness on a plate shaker (300 rpm) for 30 minutes 20°C. After washing the plates five times in washing buffer (20 mmol/L TRIS, 50 mmol/L NaCl, pH 7.2), 20 μL of sample (pre-diluted 1:2)/controls/selection peptide (RLRSSVPGVR) were added. Hundred μL of 16.7 ng/mL monoclonal antibody diluted in assay buffer was added, and the plates were incubated in darkness on a plate shaker for 20 hours at 4°C. After washing the plates five times, 130 ng/mL goat anti-mouse horseradish peroxidase (HRP)-conjugated IgG antibody (Thermo Scientific, Waltham, MA, USA) diluted in assay buffer was added to each well, and the plates were incubated in darkness on a plate shaker for 1 hour at 20°C. After washing the plates five times in washing buffer, 100 μL Tetramethylbenzidine (Kem-En-Tec Diagnostics, Taastrup, Denmark) was added and the plates were incubated in darkness on a plate shaker for 15 minutes at 20°C. The reaction was stopped using 100 μL of 1% sulfuric acid. The plates were analyzed in a VersaMax ELISA microplate reader at 450 nm with 650 nm as reference. A standard curve was plotted using a 4-parametric mathematical fit model, and data were analyzed using GraphPad Prism version 7 (GraphPad Software, Inc.).

#### Technical evaluation

To evaluate the technical performance of the VIM ELISA assay, the following validation tests were carried out: Intra-inter assay variation, linearity, lower limit of detection, upper limit of detection, analyte stability (freeze/thaw and storage) and interference. The inter-intra assay variation was determined based on ten independent runs on different days using five quality controls of known concentration covering the detection range. Three of the quality controls were serum samples and two were selection peptide diluted in buffer. The intra-assay variation was calculated as the mean coefficient of variance (CV%) within plates and the inter-assay variation was calculated as the mean CV% between the ten individual runs analyzed on different days.

#### Cleavage of vimentin

To test the specificity of the VIM antibody towards vimentin cleaved by MMP2 and MMP8, recombinant human vimentin (Novus Biologicals, CO, USA) was reconstituted to a final concentration of 1000 ug/mL in 4 mM HCl. MMP2 and 8 was added to the reconstituted vimentin 1:10 (2 µg protease and 20 µg vimentin) in MMP-buffer (50 mM Tris-HCl, 150 mM NaCl, 10 mM CaCl2, 10 µM ZnCl, 0.05%Brij35, pH 7.5). The solution was incubated at 37°C for 24 hours. Digestion of methyl transferrin was included as a positive control and MMP-buffer with only proteases was included as a negative control. The digestion was stopped by adding 1 µm EDTA to each vial. Vials were stored at –80°C until analysis. The activity of the proteases was confirmed by silver staining (SilverXpress^®^ Invitrogen, CA, USA) and Coomassie blue according to manufacturer’s instructions (data not shown). The specificity of the VIM antibody towards digested vimentin was confirmed by the VIM ELISA assay. To assess the clinical relevance of the VIM assay VIM serum levels were measured in serum samples from a small cohort of patients with different cancer diseases (cohort 1).

#### Clinical evaluation of VIM and VICM

To compare VIM with VICM serum levels they were measured in two independent cohorts of patients with stage I-IV NSCLC and measured in a cohort of COPD patients and compared to healthy controls. VICM was measured according to manufactures instructions (Nordic Bioscience A/S, Denmark) (see also [60]). VIM was measured according to above procedure.

### Statistical analysis

The nonparametric Mann-Whitney test was used to compare biomarker levels between patients and controls. To evaluate the difference between different stages of NSCLC a one-way ANOVA with Kruskal-Wallis test was used. The diagnostic accuracy of VIM and VICM was obtained from the AUROC curve. Pearson’s correlation coefficient was used to calculate the correlation between VIM and VICM. A *p*-value of *P* < 0.05 was considered statistically significant. Graph design and statistical analyses were performed using GraphPad Prism version 7 (GraphPad Software, Inc.) and MedCalc version 14 (MedCalc Software).

## SUPPLEMENTARY MATERIALS



## Abbreviations

PTMs: Post-translational modifications
EMT: Epithelial-to-mesenchymal transition
CAFs: Cancer-associated fibroblasts
MMPs: Matrix metalloproteinases
VICM: MMP-degraded citrullinated vimentin
VIM: MMP-degraded non-citrullinated vimentin
NSCLC: Non-small cell lung cancer
COPD: Chronic obstructive pulmonary disease
AUROC: Area under the receiver operating characteristics curve
CV%: Coefficient of variance
ELISA: Enzyme-linked immunosorbent assay

## References

[1] Poulet G , Massias J , Taly V . Liquid Biopsy: General Concepts. Acta Cytol. 2019; 15:1–7. 10.1159/000499337. 31091522

[2] Brücher BLDM , Jamall IS . Undervalued ubiquitous proteins. 4open. 2019; 2:7 10.1051/fopen/2019002.

[3] Willumsen N , Bager CL , Leeming DJ , Smith V , Karsdal MA , Dornan D , Bay-Jensen AC . Extracellular matrix specific protein fingerprints measured in serum can seperate pancreatic cancer patients from healthy controls. BMC Cancer. 2013; 13:554. 10.1186/1471-2407-13-554. 24261855 PMC4222497

[4] Bager CL , Willumsen N , Leeming DJ , Smith V , Karsdal MA , Dornan D , Bay-Jensen AC . Collagen degradation products measured in serum can separate ovarian and breast cancer patients from healthy controls: A preliminary study. Cancer Biomarkers. 2015; 15:783–8. 10.3233/CBM-150520. 26406420 PMC12965475

[5] Bager CL , Gudmann N , Willumsen N , Leeming DJ , Karsdal MA , Bay-Jensen AC , Høgdall E , Balslev I , He Y . Quantification of fibronectin as a method to assess *ex vivo* extracellular matrix remodeling. Biochem Biophys Res Commun. 2016; 478:586–91. 10.1016/j.bbrc.2016.07.108. 27475500

[6] Kehlet SN , Sanz-Pamplona R , Brix S , Leeming DJ , Karsdal MA , Moreno V . Excessive collagen turnover products are released during colorectal cancer progression and elevated in serum from metastatic colorectal cancer patients. Sci Rep. 2016; 6:1–7. 10.1038/srep30599. 27465284 PMC4964349

[7] Jensen C , Nielsen SH , Mortensen JH , Kjeldsen J , Klinge LG , Krag A , Harling H , Jørgensen LN , Karsdal MA , Willumsen N . Serum type XVI collagen is associated with colorectal cancer and ulcerative colitis indicating a pathological role in gastrointestinal disorders. Cancer Med. 2018; 7:4619–25. 10.1002/cam4.1692. 30030909 PMC6144245

[8] Jensen C , Madsen DH , Hansen M , Schmidt H , Svane IM , Karsdal MA , Willumsen N . Non-invasive biomarkers derived from the extracellular matrix associate with response to immune checkpoint blockade (anti-CTLA-4) in metastatic melanoma patients. J Immunother Cancer. 2018; 6:152. 10.1186/s40425-018-0474-z. 30567561 PMC6300009

[9] Thorlacius-Ussing J , Kehlet SN , Rønnow SR , Karsdal MA , Willumsen N . Non-invasive profiling of protease-specific elastin turnover in lung cancer: biomarker potential. J Cancer Res Clin Oncol. 2019; 145:383–92. 10.1007/s00432-018-2799-x. 30467633 PMC11810429

[10] Leeming DJ , Bay-Jensen AC , Vassiliadis E , Larsen MR , Henriksen K , Karsdal MA . Post-translational modifications of the extracellular matrix are key events in cancer progression: Opportunities for biochemical marker development. Biomarkers. 2011; 16:193–205. 10.3109/1354750X.2011.557440. 21506694

[11] Karsdal MA , Henriksen K , Leeming DJ , Mitchell P , Duffin K , Barascuk N , Klickstein L , Aggarwal P , Nemirovskiy O , Byrjalsen I , Qvist P , Bay-Jensen AC , Dam EB , et al. Biochemical markers and the FDA critical Path: How biomarkers may contribute to the understanding of pathophysiology and provide unique and necessary tools for drug development. Biomarkers. 2009; 14:181–202. 10.1080/13547500902777608. 19399662

[12] Pankova D , Chen Y , Terajima M , Schliekelman MJ , Baird BN , Fahrenholtz M , Sun L , Gill BJ , Vadakkan TJ , Kim MP , Ahn YH , Roybal JD , Liu X , et al. Cancer-Associated Fibroblasts Induce a Collagen Cross-link Switch in Tumor Stroma. Mol Cancer Res. 2016; 14:287–95. 10.1158/1541-7786.MCR-15-0307. 26631572 PMC4794404

[13] Slawson C , Lakshmanan T , Knapp S , Hart G . A mitotic GlcNAcylation/phosphorylation signaling complex alters the posttranslational state of the cytoskeletal protein vimentin. Mol Biol Cell. 2008; 19:4130–40. 10.1091/mbc.e07-11-1146. 18653473 PMC2555957

[14] Sipilä K , Haag S , Denessiouk K , Kap̈ylä J , Peters EC , Denesyuk A , Hansen U , Konttinen Y , Johnson MS , Holmdahl R , Heino J . Citrullination of collagen II affects integrin-mediated cell adhesion in a receptor-specific manner. FASEB J. 2014; 28:3758–68. 10.1096/fj.13-247767. 24823363

[15] Sipilä KH , Drushinin K , Rappu P , Jokinen J , Salminen TA , Salo AM , Käpyla J , Myllyharju J , Heino J . Proline hydroxylation in collagen supports integrin binding by two distinct mechanisms. J Biol Chem. 2018; 293:7645–58. 10.1074/jbc.RA118.002200. 29615493 PMC5961056

[16] Satelli A , Li S . Vimentin in cancer and its potential as a molecular target for cancer therapy. Cell Mol Life Sci. 2011; 68:3033–46. 10.1007/s00018-011-0735-1. 21637948 PMC3162105

[17] Wang L , Zhang J , Banerjee S , Barnes L , Barnes L , Sajja V , Liu Y , Guo B , Du Y , Agarwal MK , Wald DN , Wang Q , Yang J . Sumoylation of vimentin354 is associated with PIAS3 inhibition of glioma cell migration. Oncotarget. 2010; 1:620–27. 10.18632/oncotarget.196. 21317457 PMC3248133

[18] Van Steendam K , Tilleman K , De Ceuleneer M , De Keyser F , Elewaut D , Deforce D . Citrullinated vimentin as an important antigen in immune complexes from synovial fluid of rheumatoid arthritis patients with antibodies against citrullinated proteins. Arthritis Res Ther. 2010; 12:R132. 10.1186/ar3070. 20609218 PMC2945022

[19] Snider NT , Ku NO , Omary MB . The sweet side of vimentin. eLife. 2018; 7:e35336. 10.7554/eLife.35336. 29513215 PMC5841928

[20] Danielsson F , Peterson M , Caldeira Araújo H , Lautenschläger F , Gad A . Vimentin Diversity in Health and Disease. Cells. 2018; 7:147. 10.3390/cells7100147. 30248895 PMC6210396

[21] Green KJ , Böhringer M , Gocken T , Jones JCR . Intermediate filament associated proteins. Adv Protein Chem. 2005; 70:143–202. 10.1016/S0065-3233(05)70006-1. 15837516

[22] Carter V , Shenton BK , Jaques B , Turner D , Talbot D , Gupta A , Chapman CE , Matthews CJ , Cavanagh G . Vimentin antibodies: A non-HLA antibody as a potential risk factor in renal transplantation. Transplant Proc. 2005; 37:654–7. 10.1016/j.transproceed.2004.12.043. 15848491

[23] Bornheim R , Müller M , Reuter U , Herrmann H , Büssow H , Magin TM . A dominant vimentin mutant upregulates Hsp70 and the activity of the ubiquitin-proteasome system, and causes posterior cataracts in transgenic mice. J Cell Sci. 2008; 121:3737–46. 10.1242/jcs.030312. 18940912

[24] Peuhu E , Virtakoivu R , Mai A , Wärri A , Ivaska J . Epithelial vimentin plays a functional role in mammary gland development. Development. 2017; 144:4103–13. 10.1242/dev.154229. 28947532

[25] Colucci-Guyon E , Giménez YRM , Maurice T , Babinet C , Privat A . Cerebellar defect and impaired motor coordination in mice lacking vimentin. Glia. 1999; 25:33–43. 10.1002/(SICI)1098-1136(19990101)25:1<33::AID-GLIA4>3.0.CO;2-J. 9888296

[26] Nieminen M , Henttinen T , Merinen M , Marttila-Ichihara F , Eriksson JE , Jalkanen S . Vimentin function in lymphocyte adhesion and transcellular migration. Nat Cell Biol. 2006; 8:156–62. 10.1038/ncb1355. 16429129

[27] Cheng F , Shen Y , Mohanasundaram P , Lindström M , Ivaska J , Ny T , Erikss JE . Vimentin coordinates fibroblast proliferation and keratinocyte differentiation in wound healing via TGF-β-Slug signaling. Proc Natl Acad Sci U S A. 2016; 113:E4320–7. 10.1073/pnas.1519197113. 27466403 PMC4968728

[28] Eckes B , Colucci-Guyon E , Smola H , Nodder S , Babinet C , Krieg T , Martin P . Impaired wound healing in embryonic and adult mice lacking vimentin. J Cell Sci. 2000; 113:2455–62. 10852824 10.1242/jcs.113.13.2455

[29] Ivaska J , Pallari HM , Nevo J , Eriksson JE . Novel functions of vimentin in cell adhesion, migration, and signaling. Exp Cell Res. 2007; 313:2050–62. 10.1016/j.yexcr.2007.03.040. 17512929

[30] Willumsen N , Bager CL , Leeming DJ , Smith V , Christiansen C , Karsdal MA , Dornan D , Bay-Jensen AC . Serum biomarkers reflecting specific tumor tissue remodeling processes are valuable diagnostic tools for lung cancer. Cancer Med. 2014; 3:1136–45. 10.1002/cam4.303. 25044252 PMC4302665

[31] Singh S , Sadacharan S , Su S , Belldegrun A , Persad S , Singh G . Overexpression of vimentin: Role in the invasive phenotype in an androgen-independent model of prostate cancer. Cancer Res. 2003; 63:2306–11. ] 12727854

[32] Takemura K , Hirayama R , Hirokawa K , Inagaki M , Tsujimura K , Esaki Y , Mishima Y . Expression of vimentin in gastric cancer: A possible indicator for prognosis. Pathobiology. 1994; 62:149–54. 10.1159/000163895. 7945921

[33] Hu L , Lau SH , Tzang CH , Wen JM , Wang W , Xie D , Huang M , Wang Y , Wu MC , Huang JF , Zeng WF , Sham JST , Yang M , et al. Association of Vimentin overexpression and hepatocellular carcinoma metastasis. Oncogene. 2004; 23:298–302. 10.1038/sj.onc.1206483. 14647434

[34] Kokkinos MI , Wafai R , Wong MK , Newgreen DF , Thompson EW , Waltham M . Vimentin and epithelial-mesenchymal transition in human breast cancer - Observations *in vitro* and *in vivo* . Cells Tissues Organs. 2007; 185:191–203. 10.1159/000101320. 17587825

[35] Richardson AM , Havel LS , Koyen AE , Konen JM , Shupe J , Wiles WG , Martin WD , Grossniklaus HE , Sica G , Gilbert-Ross M , Marcus AI . Vimentin is required for lung adenocarcinoma metastasis via heterotypic tumor cell–Cancer-associated fibroblast interactions during collective invasion. Clin Cancer Res. 2018; 24:420–32. 10.1158/1078-0432.CCR-17-1776. 29208669 PMC5771825

[36] Nissen NI , Karsdal M , Willumsen N . Collagens and Cancer associated fibroblasts in the reactive stroma and its relation to Cancer biology. J Exp Clin Cancer Res. 2019; 38:115. 10.1186/s13046-019-1110-6. 30841909 PMC6404286

[37] Augsten M . Cancer-Associated Fibroblasts as Another Polarized Cell Type of the Tumor Microenvironment. Front Oncol. 2014; 4:1–8. 10.3389/fonc.2014.00062. 24734219 PMC3973916

[38] Liu CY , Lin HH , Tang MJ , Wang YK . Vimentin contributes to epithelial-mesenchymal transition cancer cell mechanics by mediating cytoskeletal organization and focal adhesion maturation. Oncotarget. 2015; 6:15966–83. 10.18632/oncotarget.3862. 25965826 PMC4599250

[39] Wang Z , Divanyan A , Jourd’heuil FL , Goldman RD , Ridge KM , Jourd’heuil D , Lopez-Soler RI . Vimentin expression is required for the development of EMT-related renal fibrosis following unilateral ureteral obstruction in mice. Am J Physiol Renal Physiol. 2018; 315:F769–80. 10.1152/ajprenal.00340.2017. 29631355 PMC6335003

[40] Alcover A , Molano J , Renart J , Gil-Aguado A , Nieto A , Avila J . Antibodies to vimentin intermediate filaments in sera from patients with systemic lupus erythematosus. Arthritis Rheum. 1984; 27:922–8. 10.1002/art.1780270812. 6380505

[41] Kinloch AJ , Kaiser Y , Wolfgeher D , Ai J , Eklund A , Clark MR , Grunewald J . *In situ* humoral immunity to vimentin in HLA-DRB1*03+ patients with pulmonary sarcoidosis . Front Immunol. 2018; 9:1516. 10.3389/fimmu.2018.01516. 30038611 PMC6046378

[42] Thiagarajan PS , Yakubenko VP , Elsori DH , Yadav SP , Willard B , Tan CD , Rodriguez ER , Febbraio M , Cathcart MK . Vimentin is an endogenous ligand for the pattern recognition receptor Dectin-1. Cardiovasc Res. 2013; 99:494–504. 10.1093/cvr/cvt117. 23674515 PMC3718321

[43] Li FJ , Surolia R , Li H , Wang Z , Kulkarni T , Liu G , de Andrade JA , Kass DJ , Thannickal VJ , Duncan SR , Antony VB . Autoimmunity to Vimentin Is Associated with Outcomes of Patients with Idiopathic Pulmonary Fibrosis. J Immunol. 2017; 199:1596–605. 10.4049/jimmunol.1700473. 28754682 PMC5563167

[44] Fasipe TA , Hong SH , Da Q , Valladolid C , Lahey MT , Richards LM , Dunn AK , Cruz MA , Marrelli SP . Extracellular vimentin/VWF (von Willebrand factor) interaction contributes to VWF string formation and stroke pathology. Stroke. 2018; 49:2536–40. 10.1161/STROKEAHA.118.022888. 30355099 PMC6207252

[45] Shigyo M , Kuboyama T , Sawai Y , Tada-Umezaki M , Tohda C . Extracellular vimentin interacts with insulin-like growth factor 1 receptor to promote axonal growth. Sci Rep. 2015; 5:12055. 10.1038/srep12055. 26170015 PMC4501001

[46] Shigyo M , Tohda C . Extracellular vimentin is a novel axonal growth facilitator for functional recovery in spinal cord-injured mice. Sci Rep. 2016; 6:28293. 10.1038/srep28293. 27323867 PMC4915015

[47] Walker JL , Bleaken BM , Romisher AR , Alnwibit AA , Menko AS . In wound repair vimentin mediates the transition of mesenchymal leader cells to a myofibroblast phenotype. Mol Biol Cell. 2018; 29:1555–70. 10.1091/mbc.e17-06-0364. 29718762 PMC6080657

[48] Bonotti A , Simonini S , Pantani E , Giusti L , Donadio E , Mazzoni MR , Chella A , Marconi L , Ambrosino N , Lucchi M , Mussi A , Cristaudo A , Foddis R . Serum mesothelin, osteopontin and vimentin: Useful markers for clinical monitoring of malignant pleural mesothelioma. Int J Biol Markers. 2017; 32:e126–31. 10.5301/jbm.5000229. 27646775

[49] Huet D , Bagot M , Loyaux D , Capdevielle J , Conraux L , Ferrara P , Bensussan A , Marie-Cardine A . SC5 mAb Represents a Unique Tool for the Detection of Extracellular Vimentin as a Specific Marker of Sézary Cells. J Immunol. 2006; 1:652–9. 10.4049/jimmunol.176.1.652. 16365461

[50] Li H , Qiu Z , Li F , Wang C . The relationship between MMP-2 and MMP-9 expression levels with breast cancer incidence and prognosis. Oncol Lett. 2017; 14:5865–70. 10.3892/ol.2017.6924. 29113219 PMC5661385

[51] Wang QM , Lv LI , Tang Y , Zhang LI , Wang LF . MMP-1 is overexpressed in triple-negative breast cancer tissues and the knockdown of MMP-1 expression inhibits tumor cell malignant behaviors *in vitro* . Oncol Lett. 2019; 17:1732–40. 10.3892/ol.2018.9779. 30675232 PMC6341686

[52] van Venrooij WJ , Pruijn GJM . Citrullination: A small change for a protein with great consequences for rheumatoid arthritis. Arthritis Res. 2000; 2:249–51. 10.1186/ar95. 11094435 PMC130012

[53] Makrygiannakis D , Af Klint E , Lundberg IE , Löfberg R , Ulfgren AK , Klareskog L , Catrina AI . Citrullination is an inflammation-dependent process. Ann Rheum Dis. 2006; 65:1219–22. 10.1136/ard.2005.049403. 16540548 PMC1798285

[54] Vossenaar ER , Radstake TRD , Van Der Heijden A , Van Mansum MAM , Dieteren C , De Rooij DJ , Barrera P , Zendman AJW , Van Venrooij WJ . Expression and activity of citrullinating peptidylarginine deiminase enzymes in monocytes and macrophages. Ann Rheum Dis. 2004; 63:373–81. 10.1136/ard.2003.012211. 15020330 PMC1754951

[55] Sipilä KH , Ranga V , Rappu P , Mali M , Pirilä L , Heino I , Jokinen J , Käpylä J , Johnson MS , Heino J . Joint inflammation related citrullination of functional arginines in extracellular proteins. Sci Rep. 2017; 7:8246. 10.1038/s41598-017-08597-4. 28811641 PMC5557964

[56] Mortensen JH , Manon-Jensen T , Jensen MD , Hägglund P , Klinge LG , Kjeldsen J , Krag A , Karsdal MA , Bay-Jensen AC . Ulcerative colitis, Crohn’s disease, and irritable bowel syndrome have different profiles of extracellular matrix turnover, which also reflects disease activity in Crohn’s disease. PLoS One. 2017; 12:e0185855. 10.1371/journal.pone.0185855. 29028807 PMC5640222

[57] Bay-Jensen AC , Karsdal MA , Vassiliadis E , Wichuk S , Marcher-Mikkelsen K , Lories R , Christiansen C , Maksymowych WP . Circulating citrullinated vimentin fragments reflect disease burden in ankylosing spondylitis and have prognostic capacity for radiographic progression. Arthritis Rheum. 2013; 65:972–80. 10.1002/art.37843. 23280360

[58] Mortensen JH , Godskesen LE , Jensen MD , Van Haaften WT , Klinge LG , Olinga P , Dijkstra G , Kjeldsen J , Karsdal MA , Bay-Jensen AC , Krag A . Fragments of Citrullinated and MMP-degraded Vimentin and MMP-degraded Type III Collagen Are Novel Serological Biomarkers to Differentiate Crohn’s Disease from Ulcerative Colitis. J Crohns Colitis. 2015; 9:863–72. 10.1093/ecco-jcc/jjv123. 26188349

[59] Siebuhr AS , Juhl P , Bay-Jensen AC , Karsdal MA , Franchimont N , Chavez JC . Citrullinated vimentin and biglycan protein fingerprints as candidate serological biomarkers for disease activity in systemic sclerosis: a pilot study. Biomarkers. 2018; 24:249–54. 10.1080/1354750X.2018.1548032. 30457356

[60] Vassiliadis E , Oliveira CP , Alvares-da-Silva MR , Zhang C , Carrilho FJ , Stefano JT , Rabelo F , Pereira L , Kappel CR , Henriksen K , Veidal SS , Vainer B , Duffin KL , et al. Circulating levels of citrullinated and MMP-degraded vimentin (VICM) in liver fibrosis related pathology. Am J Transl Res. 2012; 4:403–14. 23145208 PMC3493028

[61] Lugli EB , Correia RE , Fischer R , Lundberg K , Bracke KR , Montgomery AB , Kessler BM , Brusselle GG , Venables PJ . Expression of citrulline and homocitrulline residues in the lungs of non-smokers and smokers: implications for autoimmunity in rheumatoid arthritis. Arthritis Res Ther. 2015; 17:9. 10.1186/s13075-015-0520-x. 25600626 PMC4349479

[62] Mortensen JH , Guo X , De Los Reyes M , Dziegiel MH , Karsdal MA , Bay-Jensen AC , White WI . The VICM biomarker is released from activated macrophages and inhibited by anti-GM-CSFRα-mAb treatment in rheumatoid arthritis patients. Clin Exp Rheumatol. 2019; 37:73–80. 30418117

[63] Kidd ME , Shumaker DK , Ridge KM . The role of Vimentin intermediate filaments in the progression of lung cancer. Am J Respir Cell Mol Biol. 2014; 50:1–6. 10.1165/rcmb.2013-0314TR. 23980547 PMC3930939

[64] American Cancer Society. Non-Small Cell Lung Cancer Stages. 2017 https://www.cancer.org/cancer/non-small-cell-lung-cancer/detection-diagnosis-staging/staging.html.

[65] Al-Saad S , Al-Shibli K , Donnem T , Persson M , Bremnes RM , Busund LT . The prognostic impact of NF-κB p105, vimentin, E-cadherin and Par6 expression in epithelial and stromal compartment in non-small-cell lung cancer. Br J Cancer. 2008; 99:1476–83. 10.1038/sj.bjc.6604713. 18854838 PMC2579693

[66] Richardson F , Young GD , Sennello R , Wolf J , Argast GM , Mercado P , Davies A , Epstein DM , Wacker B . The evaluation of E-cadherin and vimentin as biomarkers of clinical outcomes among patients with non-small cell lung cancer treated with erlotinib as second- or third-line therapy. Anticancer Res. 2012; 32:537–52. 22287743

[67] Adam SA , Geracet L . Cytosolic proteins that specifically bind nuclear location signals are receptors for nuclear import. Cell. 1991; 66:837–47. 10.1016/0092-8674(91)90431-W. 1653647

[68] Combet C , Blanchet C , Geourjon C , Deléage G . NPS@: Network Protein Sequence Analysis. Trends Biochem Sci. 2000; 25:147–50. 10.1016/s0968-0004(99)01540-6. 10694887

