# Supplementary material for: Post-translational modifications of vimentin reflect different pathological processes associated with non-small cell lung cancer and chronic obstructive pulmonary disease
Source: Oncotarget. 2019 Nov 26;10(63):6829–41. doi: 10.18632/oncotarget.27332 (PMC6887574; doi:10.18632/oncotarget.27332)
Supplement: Supplementary file 1 [file oncotarget-10-6829-s001.pdf]

Post-translational modifications of vimentin reflect different pathological processes associated with non-small cell lung cancer and chronic obstructive pulmonary disease

SUPPLEMENTARY MATERIALS

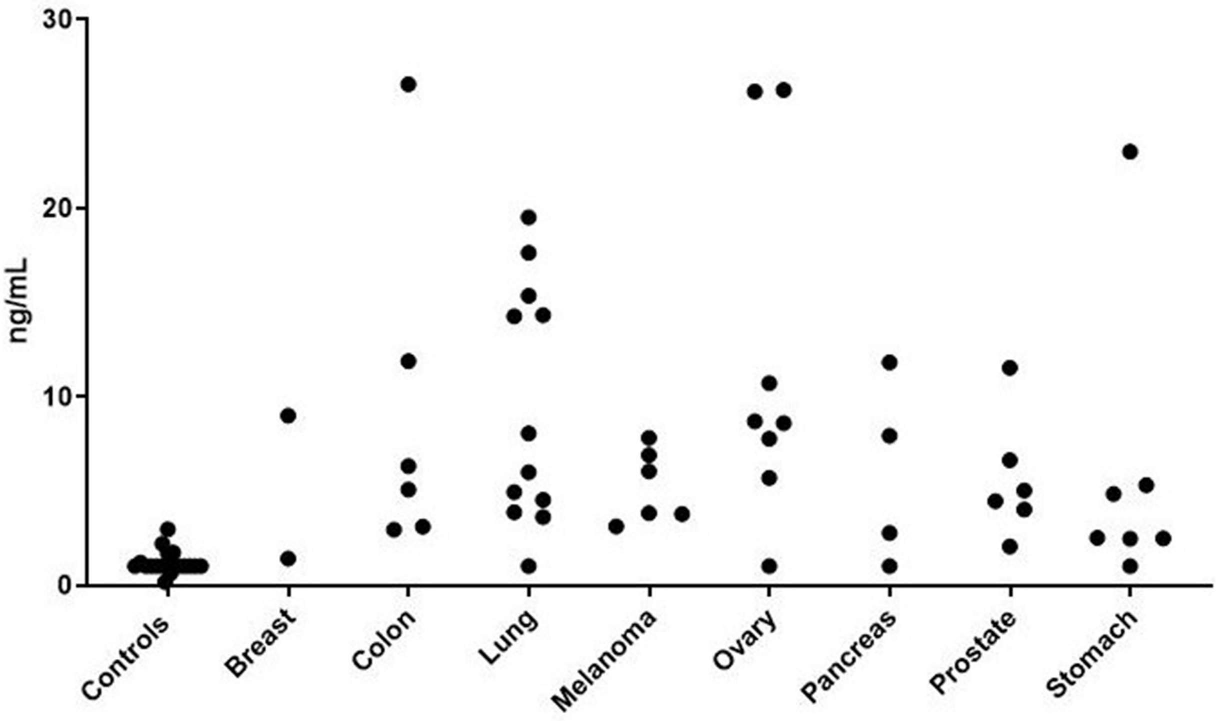

Supplementary Figure 1: Validation of the MMP-degraded non-citrullinated vimentin (VIM) assay in serum from patients with various solid tumors. VIM was assessed in serum from healthy controls (n = 19) and in serum from patients with solid tumors; breast (n = 2), colon (n = 3), lung (n = 12), malignant melanoma (n = 6), ovarian (n = 8), pancreas (n = 4), prostate (n = 6), stomach cancer (n = 7).
